# Paeonidin- and Cyanidin-Rich Fractions Ameliorate Gut Microbiota and Serum Metabolites in Rats with Type 2 Diabetes

**DOI:** 10.3390/nu18213520

**Published:** 2026-10-26

**Authors:** Chenchen Li, Shuying Zhao, Wei Mi, Zhiyong Hu, Tala Shi

**Affiliations:** School of Public Health, Shandong Medical and Pharmaceutical University, Yantai 264003, China

**Keywords:** paeonidin, cyanidin, type 2 diabetes mellitus, gut microbiota, serum metabolites

## Abstract

Background: Dietary intervention could modulate gut microbiota, improve insulin resistance status, and play a key role in blood glucose regulation. Objective: This study investigated the regulatory effects of paeonidin- and cyanidin-rich fractions (PCs) on gut microbiota and serum metabolites in high-fat, high-sucrose diet/streptozotocin-induced type 2 diabetes mellitus (T2DM) rats. Method: Following a 14-day intragastric PC administration (25 mg/kg/day), non-targeted metabolomics and 16S rDNA sequencing were performed. Results: PC intervention significantly reduced water intake, and even the effects on lipids were limited. Among 83 differential serum metabolites, PC restored 28, including tauroursodeoxycholic acid (TUDCA) and lysophosphatidylcholines, while decreasing glycated branched-chain amino acid derivatives. PC-enriched *Bifidobacterium*, *Sediminibacterium*, *SC-I-84*, and *Fusicatenibacter*, which positively correlated with TUDCA. Additionally, PC upregulated insulin secretion and p53 signaling pathways with increased RYR2 and FAS, while downregulating pentose and glucuronate interconversion enzymes (UxaC, XylB). Conclusions: These findings suggest that the amelioration of glycolipid metabolic disorders and insulin secretion defects by PC may be associated with a synergistic gut microbiota-metabolite-enzyme axis, potentially involving *Bifidobacterium*-mediated TUDCA elevation and downstream modulation of metabolic enzymes.

## 1. Introduction

Type 2 diabetes mellitus (T2DM) accounts for approximately 90% of all diabetic cases and represents a major public health burden. Given its complex pathophysiology, T2DM often requires combination therapies. However, challenges such as adverse effects, secondary failure, and high costs persist, highlighting the need for better-tolerated, multi-targeted strategies, particularly those derived from natural products [1,2].

The Takayama study suggested a protective effect of dietary polyphenols against diabetes, showing that subjects in the highest quartile of total polyphenol intake were more likely to have no history of diabetes [3]. Purple sweet potato anthocyanin (PSPA) is characterized by excellent stability to light and heat, with the primary anthocyanin monomers including paeonidin (PD), cyanidin (CD), and delphinidin [4]. In our previous study, we found that PD is the main component of PSPA, which could decrease the fasting blood glucose level and regulate the gut microbiota of T2DM mice [5]. Another study also identified that PD-based components in PSPA showed good properties in scavenging superoxide anions and 1,1-diphenyl-2-picrylhydrazyl radicals, as well as strong ferric reducing antioxidant power and Fe^2+^ chelating ability [6]. Moreover, the PD combined with other anthocyanin extracts could mitigate T2DM-related indices, such as hyperglycemia and oxidative stress, through multiple pathways. As major components of black soybean seed coat extract, the cyanidin-3-O-glucoside (C3G), delphinidin-3-O-glucoside (D3G), and peonidin-3-O-glucoside (P3G) treatments remarkably decreased the serum insulin levels of diabetic mice, exhibited strong inhibitory activity for α-amylase, and demonstrated potential in inhibiting lipid accumulation in HepG2 cells, while also protecting HepG2 cells from H_2_O_2_-induced oxidative stress [7]. The anthocyanin fraction rich in C3G and P3G counteracted nuclear factor kappa-light-chain-enhancer of activated B (NF-κB) cells activity and the NOD-like receptor family pyrin domain containing three (NLRP3) inflammatory pathways, leading to the inhibition of inflammatory genes and cytokine secretions in lung epithelial cells and activated macrophages [8]. Su et al. confirmed the synergistic inhibition of pentapeptide and anthocyanin-paeonidin against dipeptidyl peptidase-IV (DPP-IV), a metabolic enzyme that degrades and inactivates glucagon-like peptide-1 (GLP-1) and glucagon-dependent insulinotropic polypeptide (GIP) by cleaving their *N*-terminal dipeptide residues. GLP-1 and GIP are incretins that promote insulin secretion and inhibit glucagon secretion in response to glucose stimulation, thereby exerting a glucose-regulating effect [9,10].

Furthermore, research indicates significant differences in blood metabolite concentrations between T2DM patients and non-diabetic controls. The Framingham offspring study identified elevated levels of branched-chain amino acids (BCAAs—isoleucine, leucine, and valine) and aromatic amino acids (tyrosine and phenylalanine) as strong predictors of T2DM [11]. A nested case-control study by Lu et al. [12] involving 197 individuals found significantly elevated baseline levels of BCAAs, non-esterified fatty acids (palmitic, stearic, oleic, and linoleic acids), carbohydrates (CDP-glucose, D-galactose, and gluconate), and organic acids (acetoacetate, lactate, pyruvate, and urea) in cases. In contrast, proline, serine, and medium-chain acylcarnitines (C5, C8, C10, and C13) were significantly reduced. Compared to controls, cases also exhibited alterations in lysophospholipids, including increased lysophosphatidylinositol (LPI) and lysophosphatidylglycerol (LPG), and decreased lysophosphatidylcholine (LPC) and lysophosphatidylethanolamine (LPE). Lipid metabolism disorders in T2DM may also be linked to gut microbiota [13]. T2DM patients exhibit significantly reduced gut microbial diversity, and complex interactions between the gut microbiota and the intestinal mucosa may play a critical role in the pathogenesis of T2DM. 16S rRNA gene sequencing has shown markedly decreased gut microbiota diversity in diabetic patients compared to healthy individuals, with significant reductions in butyrate-producing bacteria such as *Bifidobacterium* and *Akkermansia*. Furthermore, the abundance of *Dorea* is significantly increased in T2DM individuals [14]. Gut bacteria such as *Lactobacillus* and *Bifidobacterium*, which exhibit β-glucosidase activity, can convert anthocyanins into more bioavailable forms [15]. Anthocyanins possess prebiotic potential, interacting with the gut microbiota to produce beneficial metabolites and modulate microbial composition [16,17]. However, the regulatory effects of polyacylated anthocyanins on gut microbiota and their metabolites in T2DM have not been reported in vivo.

To address this gap, this present study investigates the effect of a PD- and CD-enriched anthocyanin fraction on gut microbiota and blood metabolite profiles in a T2DM rat model, integrating microbiome and metabolomic analyses to elucidate the mechanisms underlying its glucose-lowering activity. This work aims to provide new insights into the antidiabetic potential of PD and CD-based anthocyanins and to support their future application as functional food ingredients.

## 2. Materials and Methods

### 2.1. Animal Model and Intervention Method

The T2DM rat model was established based on a previously described method [7]. Twenty-five male Sprague-Dawley (SD) rats (180–200 g, purchased from Jinan Pengyue, Shandong, China, certified to be free of specific pathogens) were housed in a pathogen-free facility under controlled conditions: 25 ± 3 °C, 55 ± 5% relative humidity, and a 12-h light/12-h dark cycle, with ad libitum access to water and diet. Following a one-week acclimatization, the rats were randomly divided into the control group (C, *n* = 5) and model group (M, *n* = 20, 2–3 rats/cage). The C group was fed a standard diet (50% basal feed, 5% soybean meal, 5% casein, 10% milk powder, 5% peanuts, 5% egg yolk powder, 12% lard, 5% sucrose, 1% sesame oil, 2% salt, and 10 drops of vitamin A). The M group was fed high-fat, high-sucrose (HFHS) diets (Purchased from Beijing Keao Xieli Feed Co., Ltd., Beijing, China, which was formulated by supplementing the basal feed with 10% lard, 20% sucrose, 2.5% cholesterol, and 0.5% sodium cholate). The blood glucose levels and body weight were monitored weekly to confirm the establishment of the diabetic model. After four weeks, the rats were fasted for 12 h; the M group received a single intraperitoneal injection of freshly prepared streptozotocin (STZ, 50 mg/kg in 0.1 mol/L citrate buffer, pH 4.5), and the C group received an equivalent volume of citrate buffer. After 72 h, fasting blood glucose (FBG) levels were measured, and rats with FBG levels ≥ 11.1 mmol/L were considered to be the T2DM model. The successfully modeled 12 rats were further divided into the model group (M, *n* = 4), metformin group (MF, *n* = 3, 150 mg/kg/day metformin), and paeonidin and cyanidin fractions group (PC, *n* = 5, 25 mg/kg/day), according to body weight. The PC contained five kinds of PD and four kinds of CD-based anthocyanin mixture, which were extracted from purple sweet potato, with purity ≥ 90%, and purchased from Caoyuankang Biotechnology Co., Ltd. (Chengdu, Sichuan, China). The high-performance liquid chromatography (HPLC) compositional characteristics and peak area of PC are shown in Figure 1A,B. The C group remained on the standard diet; the MF and PC were dissolved in saline solution and administered once daily via oral gavage. The C and M groups received an equivalent volume of saline (0.5 mL/rat/day). After a 14-day intervention, all rats were anesthetized by inhalation of 3–5% ether (2–3 cotton balls immerse in analytical grade ether, place them at the bottom of a transparent sealed anesthesia chamber, and put the mouse into the chamber, tighten the lid, wait for 1–2 min); when the mouse corneal reflex disappears or unable to turn over by itself, the rats were removed and the blood samples were collected via orbital; serum was separated by centrifuge at 4000× *g* for 15 min at 4 °C, and then stored at −20 °C. The liver tissues were fixed in 4% paraformaldehyde for histopathological examination. Fecal contents were aseptically collected, transferred to sterile micro-tubes, and stored at −80 °C for 16S rDNA sequencing.

### 2.2. Serum Biochemical Indexes and Metabolic Analysis

For biochemical analysis, serum glucose was measured using a glucose oxidase–peroxidase (GOPOD) colorimetric assay kit, and the results are reported as fasting serum glucose. Serum insulin (INS), low-density lipoprotein cholesterol (LDL-C), high-density lipoprotein cholesterol (HDL-C), triglyceride (TG), and total cholesterol (T-CHO) were determined using ELISA kits (purchased from Jianglai Biotechenology Co., Ltd., Shanghai, China), strictly according to the manufacturers’ instructions.

Serum untargeted metabolic analysis was performed by OE Biotechnology. A 100 μL aliquot of each serum sample was mixed with 300 μL of ice-cold methanol/acetonitrile (2:1, *v*/*v*) containing *L*-2-chlorophenylalanine (0.3 mg/mL) as an internal standard. The mixture was vortexed, sonicated in an ice-water bath for 10 min, and then incubated at −20 °C for 30 min to precipitate proteins. After centrifugation at 13,000× *g* for 10 min at 4 °C, the supernatant was collected, concentrated, and reconstituted in 100 μL of methanol/water (1:4, *v*/*v*). The solutions were centrifuged (13,000× *g* for 10 min at 4 °C) again, and the supernatant was filtered through a 0.22 μm membrane and transferred into LC vials for analysis. A quality control (QC) sample was prepared by pooling equal volumes of all individual samples. The metabolite profiling was performed using an ACQUITY UPLC I-Class system (Waters Corporation, Milford, MA, USA) coupled to a VION IMS QTOF mass spectrometer (Waters Corporation, Milford, MA, USA). Chromatographic separation was achieved on a HSS T3 C18 column (100 mm × 2.1 mm, 1.8 μm) maintained at 45 °C. The mobile phase consisted of (A) 0.1% formic acid in water and (B) acetonitrile/methanol (2:3, *v*/*v*). Mass spectrometer detection was carried out in both positive and negative electrospray ionization (ESI^+^ and ESI^−^) modes using MSE acquisition with low (4 eV) and ramped high (20–45 eV) collision energies. The mass scan range was *m*/*z* 50–1000. The raw liquid chromatography-mass spectrometer (LC-MS) data were processed for peak picking, alignment, and normalization. Metabolite identification was conducted by querying the Human Metabolome Database (HMDB), LipidMaps (V2.3), and Metlin databases. Peaks with more than 50% missing values across all samples were excluded. Orthogonal projections to latent structures-discriminant analysis (OPLS-DA) was performed to identify differentially abundant metabolites based on variable importance in projection (VIP) scores (>1.0) and univariate Student’s *t*-tests (*p* < 0.05).

### 2.3. 16S rDNA Sequencing of Gut Microbiota

Genomic DNA was extracted from fecal samples using a MagPure Soil DNA LQ Kit (Magen Biotechnology Co., Ltd., Guangzhou, China) commercial soil DNA extraction kit, following the manufacturer’s protocol. The hypervariable V3–V4 region of the bacterial 16S rRNA gene was amplified using the barcoded primers 343F and 798R. The resulting PCR products were purified, quantified, pooled in equimolar ratios, and subjected to paired-end sequencing on an Illumina MiSeq platform (Illumina, San Diego, CA, USA) according to standard protocols.

### 2.4. Statistical Analysis

All quantitative data are presented as mean ± standard deviation (SD). One-way analysis of variance (ANOVA) with Tukey’s honestly significant difference comparison. Differences between the gut microbial and serum metabolites were assessed using one-way ANOVA or the Kruskal–Wallis (KW) rank-sum test, and Dunn’s test analysis (R package) was conducted to assess differences between the two groups. Spearman’s correlation analysis was conducted using the R package (v4.0.0), and a *p*-value of less than 0.05 was considered statistically significant for all tests.

## 3. Results

### 3.1. PC Effect on Body Weight and Water Intake

As shown in Figure 1C, compared with the C group (416.96 ± 12.44 g/rat), the body weights of the M (365.68 ± 6.22 g/rat), MF (360.97 ± 27.38 g/rat), and PC (372.96 ± 7.89 g/rat) groups were significantly decreased at the end of the experimental period (*p* < 0.05). However, no significant differences were observed among the M, MF, and PC groups, indicating that PC intervention did not significantly affect body weight. However, average daily water intake during the experimental period was significantly higher in the M (92.25 ± 7.43 mL/day/rat), MF (99.58 ± 1.70 mL/day/rat), and PC (86.04 ± 5.77 mL/day/rat) groups compared to the C group (44.55 ± 1.97 mL/day/rat) (*p* < 0.05). The PC group showed significantly reduced water intake compared with the M group (*p* = 0.026, 95% CI = −25.09 to −1.99) (Figure 1D). Together, the body weight and water intake data indicate that the T2DM rat model was successfully established.

### 3.2. PC Effect on Blood Glucose and Lipid Biomarkers

The serum GOPOD, insulin, TC, TG, LDL-C, and HDL-C levels are shown in Figure 1. Blood glucose concentrations in the C group (1.61 ± 0.41 mmol/L) were significantly lower than those in the M (2.59 ± 0.45 mmol/L), MF (3.02 ± 0.21 mmol/L), and PC (2.72 ± 0.21 mmol/L) groups. However, neither the MF group nor the PC group showed a statistically significant reduction in blood glucose compared with the model group. For lipid biomarkers, the concentrations of TC, TG, and LDL-C in group C were significantly lower than those in group M; compared with group M, both the MF and PC groups exhibited a decreasing trend in these lipid parameters. In addition, the MF group showed significantly elevated HDL-C levels relative to group M, but PC intervention exerted no significant effects on the lipid profiles under our experimental condition (Figure 1C–J).

### 3.3. PC Effects on Serum Metabolites

Non-targeted metabolomic analysis shows a converged distribution of quality control (QC) samples, and a significant divergence between four groups was demonstrated by PCA. These data indicated that the instrument was highly stable and repeatable (Figure 2A). The OPLS-DA results revealed that samples from the same group were clustered together, and different groups were well distinguished. These results indicated that establishing the T2DM rats caused significant metabolic changes (Figure 2B). After aligning the MS/MS data peaks and removing the missing values, significantly different metabolites were identified using the criteria of variable importance in projection (VIP) > 1 and *p* < 0.05 (Student’s *t*-test). A total of 302 (190 up, 112 down), 153 (78 up, 75 down), 298 (184 up, 114 down), 116 (59 up, 57 down), and 83 (51 up, 32 down) metabolites were different between C and PC, MF and PC, C and M, MF and M, PC and M groups, respectively (Figure 2D,E). The number of overlapping metabolites across groups and their annotation information are presented in Figure 2C and Supplementary Table S2. Among 83 significantly different metabolites, 28 were also different between the C and M groups; compared between C and M groups, the blood LysoPC(15:0/0:0), LysoPC(22:4(7Z,10Z,13Z,16Z)/0:0), LysoPC(P-16:0/0:0), 2-[(3alpha,7alpha,12alpha-trihydroxy-24-oxocholane-24-yl)amino]ethanesulfonic acid (ESA), PC(18:0(11cp)/0:0), tauroursodeoxycholic acid (TUDCA), and TG(16:0/14:1(9Z)/22:0) decreased, but the MF and PC intervention restored those changes, with the PC increasing 74.4%, 66.6%, 78.0%, 73.9%, 41.1%, and 20.7%, respectively, except for TG(16:0/14:1(9Z)/22:0). Compared with group C, the PC group showed significantly lower levels of TG(18:0/18:0/18:2(9Z,12Z))[iso3], TG(16:0/14:1(9Z)/22:0), PC(18:0(11cp)/0:0), LysoPC(P-16:0/0:0), PE(19:0/20:2(11Z,14Z)), LysoPC(22:4(7Z,10Z,13Z,16Z)/0:0), and LysoPC(15:0/0:0). Compared with group MF, the PC group showed significantly higher levels of ESA and TUDCA, but significantly lower levels of TG (16:0/14:1(9Z)/22:0), LysoPE(0:0/22:6(4Z,7Z,10Z,13Z,16Z,19Z)), *N*-(1-deoxy-1-fructosyl), phenylalanine, and hexazinone. The blood metabolic analysis revealed obvious metabolic disorders in the model group; both MF and PC intervention ameliorated these abnormalities, suggesting a potential protective effect of PC against T2DM-induced metabolic dysregulation (Figure 2F).

### 3.4. PC Effect on Gut Microbial Composition

Alpha diversity indices were calculated at a uniform sequencing depth, and the values were plotted for each group. The *t*-test comparisons between groups revealed significant differences in Chao1 and ACE indices between the C and M groups, indicating that T2DM modulated gut microbial alpha diversity (Figure 3A,B). The PD whole tree diagram showed significant differences between the C and M groups and between the C and MF groups, but there was no significant difference observed between the C and PC groups, suggesting that PC intervention influences T2DM rat gut microbial phylogenetic diversity (Figure 3C). The binary_jaccard (R = 0.67 and *p* < 0.05) and bray_curtis (R = 0.51 and *p* < 0.05) beta diversity analyses demonstrated the differences in gut microbial composition and abundance across the groups (Figure 3D,E). Venn diagram analysis identified 21, 4, 5, and 11 group-specific genera in the C, M, MF, and PC groups, respectively (Figure 3F). The group-specific genera are listed in Supplementary Table S1.

The Dunn test significance analysis between groups at the genus level showed that 37 bacterial genera demonstrated variations between groups, as presented in Figure 3F–H. Compared with C, the M group decreased the abundance of *UBA1819* (87.9%), *Family_XIII_UCG-001* (100%), *RF39* (93.8%), *Acinetobacter* (78.5%), *F082* (77.3%), *Lachnospiraceae_UCG-001* (73.5%), *Roseburia* (70.3%), *Romboutsia* (65.9%), *Parabacteroides* (59.4%), *uncultured* (54.7%), *Parabacteroides* (59.4%), *Ruminococcaceae* (54.6%) and *Bifidobacterium* (47.1%). But compared with M, PC intervention not only restores the genus *Bifidobacterium* (53.4%) abundance, but also significantly increases the *Sediminibacterium* (100%), *Fusicatenibacter* (100%), and *SC-I-84* (61.9%), while the *Mucispirillum* (76.6%), *Incertae_Sedis* (86.6%), *A2* (100%), *Desulfovibrio* (72.6%), and *Peptococcus* (81.7%) abundances were decreased by PC. Moreover, compared with C, the M group increased the abundance of *Turicibacter* (100%), *Klebsiella* (100%), *Clostridium_sensu_stricto_1* (98.3%), *Escherichia-Shigella* (90.5%), *Treponema* (84.3%), *Streptococcus* (89.1%) and *UCG-005* (67.0%), but compared with the M group, MF intervention could significantly decrease the *Anaerovorax* (100%), *DNF00809* (100%), *UCG-005* (87.6%), *Alloprevotella* (65.7%), *Clostridia_UCG-014* (80.6%), *NK4A214_group* (77.9%), *Romboutsia* (68.1%), and *Christensenellaceae_R-7_group* (90.3%), and increase the *Prevotella* (94.8%) and *Coprococcus* (100%) abundances. Compared with the C group, the PC group still maintained significantly higher abundances of *Escherichia-Shigella* (8.3%) and *Clostridium_sensu_stricto_1* (2.3%), whereas the abundances of *Lachnospiraceae_UCG-001* (12.4%), *F082* (8.2%), and *Mucispirillum* (1.3%) were significantly lower. The PC group showed significantly increased relative abundances of *Alloprevotella* (34.5%), *UCG-005* (18.6%), *NK4A214_group* (19.7%), *Christensenellaceae_R-7_group* (16.3%), and *Romboutsia* (34.3%), but significantly decreased relative abundances of *Klebsiella* (8.6%), *Mucispirillum* (11.9%), and *Desulfovibrio* (36.5%) when compared with the MF group. Results indicate that the PC and MF had a specific effect on the T2DM rat gut microbial genera.

The prediction of metabolic pathway analysis shows that there were 42, 22, and 63 pathways that were significantly different between C and M, M and MF, and M and PC, respectively. Among them, 21 pathways were related to T2DM according to previously published literature. Compared with C, the M group had significantly increased abundance of pentose and glucuronate interconversions, type II diabetes mellitus, adrenergic signaling in cardiomyocytes, apelin signaling pathway, calcium signaling pathway, but decreased *N*-glycan biosynthesis, p53 signaling pathway, ECM-receptor interaction, and hematopoietic cell lineage. But compared with M, the MF group could increase the renin-angiotensin system but decrease the calcium signaling pathway, adrenergic signaling in cardiomyocytes, inositol phosphate metabolism, insulin resistance, insulin signaling pathway, and thyroid hormone signaling pathway, whereas PC intervention decreased the AMP-activated protein kinase signaling pathway, glucagon signaling pathway, HIF-1 signaling pathway, inositol phosphate metabolism, phosphatidylinositol signaling system, thyroid hormone signaling pathway, and increased apoptosis, insulin secretion, ras signaling pathway, and p53 signaling pathway (Figure 4A).

Further prediction on the significant different genes/enzymes analysis shows that compared with M, PC increased three enzymes, including K04962 (RYR2; ryanodine receptor 2) abundance, which annotated on calcium signaling pathway, insulin secretion and apelin signaling pathway, K11533 (FAS; fatty acid synthase, bacteria type [EC:2.3.1.-]), annotated on insulin resistance, and K01623 (ALDO; fructose-bisphosphate aldolase, class I [EC:4.1.2.13]) annotated on HIF-1signaling pathway, but had decreased abundance of enzymes K01812 (uxaC; glucuronate isomerase [EC:5.3.1.12]), and K00854 (XYLB; xylulokinase [EC:2.7.1.17]), which are found on pentose and glucuronate interconversions pathway (Figure 4B–E). To verify the prediction analysis, we used ELISA kits to test the serum RYR2, and found consistence increased content in serum samples (Figure 4F).

### 3.5. Correlation Analysis

The 37 differential bacterial genera and the 28 differential metabolites correlation analysis shows that the increased bacterial *SC-I-84* was positively associated with increased levels of TUDCA and LysoPC(P-16:0/0:0), whereas the genera *Clostridium_sensu_stricto_1*, *Klebsiella*, *Escherichia-Shigella*, and *Streptococcus* were negatively related with them. In contrast, the decreased bacterial genera *Mucispirillum*, *Desulfovibrio*, and *Incertae_Sedis* in the PC group were positively related to TG (16:0/14:1(9Z)/22:0) (Figure 5A).

Furthermore, *SC-I-84* and *Bifidobacterium* were positively correlated with K11533 (FAS; fatty acid synthase, bacteria type [EC:2.3.1.-]), and negatively correlated with K00854 (XYLB; xylulokinase [EC:2.7.1.17]), while the harmful genera were not significantly related to those enzymes. Changes in these enzyme-related features were positively associated with the type 2 diabetes mellitus pathway (Figure 5B,C). These findings suggest that PC intervention was associated with increased abundance of *SC-I-84* and *Bifidobacterium*, and that these microbial and metabolic changes may be linked to pathways related to insulin resistance, pentose and glucuronate interconversions, calcium signaling, and insulin secretion.

## 4. Discussion

The present study found that PC treatment was associated with significantly reduced water consumption of STZ-HFHS diet-induced T2DM rats. However, the effects on TC and HDL-C were less consistent: TC showed a decreasing tendency in the PC group compared with the model group, but this change did not reach statistical significance, whereas HDL-C did not show a clear improvement. These lipid changes followed broadly similar trends to those observed in the MF group, but should not be interpreted as evidence that PC modulates serum TC through the same mechanisms as MF. To explore potential mechanisms underlying these observations, we further examined the changes in gut microbiota and its metabolites after PC treatment. Among the commonly and consistently reported findings, the genera of *Bifidobacterium*, *Bacteroides*, *Faecalibacterium*, *Akkermansia*, and *Roseburia* were negatively correlated with T2DM, while the genera of *Ruminococcus*, *Fusobacterium*, and *Blautia* were positively associated with T2DM patients [18]. In our experimental animal model, we also found that the bacterial genera, including *Bifidobacterium* and *Roseburia*, were decreased in the M group, whereas the abundance of harmful genera, including *Streptococcus*, *Clostridium_sensu_stricto_1*, and *Escherichia-Shigella*, was increased. The intervention of PSPA-based PC fractions or MF showed potential in correcting disruptions in gut microbiota composition. The PC mainly elevated beneficial bacteria, whereas MF intervention specifically decreased harmful bacterial genera. The MF- and PC-specific effects on gut bacterial genera could contribute to increased glucose uptake, better lipid metabolism, and pancreatic function [19,20,21]. In vitro prebiotic activity of five peonidin-based anthocyanins also showed increased *Bifidobacterium* and *Lactobacillus* species, while inhibiting the growth of *Staphylococcus* and *Salmonella* [5]. Increased abundance of *Bifidobacterium* could increase the production of SCFAs [22], which improve glucose tolerance and reduce inflammation [23,24], thereby indirectly improving insulin sensitivity and intestinal barrier function [25,26,27]. *Fusicatenibacter* correlates with reduced insulin resistance by producing butyric acid to enhance the intestinal barrier and inhibit the TLR4/NF-κB inflammatory pathway [28]. Although the roles of *SC-I-84* and *Sediminibacterium* were not yet well studied, they were also enriched in our model. In contrast, the harmful genus *Escherichia coli* could secrete the metabolic enzyme StcE, which cleaves mucin and disrupts the intestinal barrier, and emphasize T2DM [29], whereas the *Klebsiella* genera were positively correlated with hyperglycemic states in diabetic hosts [30].

Extensive research has demonstrated that gut microbiota significantly influences T2DM via their metabolic byproducts. The gut microbiota metabolize primary bile acids into secondary bile acids, which activate host receptors (Farnesoid X Receptor (FXR), Takeda G Protein-coupled Receptor 5 (TGR5)) and influence glucose metabolism, e.g., by stimulating glucagon-like peptide-1 (GLP-1) secretion [31]. Consistent with earlier research, the present investigation confirms that PC intervention reversed 28 out of 83 differentially regulated metabolites, including bile acids (e.g., ESA, TUDCA) and LysoPCs (e.g., LysoPC(15:0/0:0), LysoPC(22:4/0:0)). TUDCA, a principal component of bile acid, has the ability to regulate insulin secretion and improve hyperglycemia [32,33,34]. Xiao et al. [35] found that two conjugated BAs (TDCA and TUDCA) could decrease body weight and fasting glucose levels in a ketogenic diet model; however, in the antibiotic cocktail-treated model, the serum levels of TDCA and TUDCA were unchanged, emphasizing the essential role of gut microbiota in regulating serum TUDCA levels. Huan et al. [36] showed that TUDCA can reduce liver fat accumulation by inhibiting FXR, increasing *Bifidobacterium* abundance, and modulating bile acid metabolism in mice with NAFLD. Cui et al. [37] found that TUDCA downregulated serum levels of TC, TG, and LDL-C, and upregulated HDL-C in hyperlipidemic mice, reduced hepatocellular fat deposition, and reversed hepatocellular steatosis, which was consistent with serum biochemical index results in our study. In our correlation analysis, TUDCA was positively related to beneficial bacteria (e.g., *SC-I-84*, *Bifidobacterium*), which may regulate blood glucose and serum TUDCA levels through the transformation between unconjugated and conjugated forms of BAs. Furthermore, LysoPCs are bioactive lipids linked to insulin resistance and inflammation [38]; their restoration by PC suggests improved membrane integrity and reduced lipotoxicity. Newgard et al. [39] found differences in plasma concentrations of branched-chain amino acids (leucine, valine), aromatic amino acids (phenylalanine), and C5 acylcarnitines between obese and lean subjects. Amino acids, peptides, and analogues, including *N*-(1-Deoxy-1-fructosyl) phenylalanine, *N*-(1-Deoxy-1-fructosyl) valine, and *N*-(1-Deoxy-1-fructosyl) leucine, are key intermediates in non-enzymatic glycation reactions between proteins/amino acids and reducing sugars. Metabolic studies identified *N*-(1-deoxy-1-fructosyl) leucine and *N*-(1-deoxy-1-fructosyl) phenylalanine, with increased levels in the extracellular fluid of HK-2 cells, which play significant roles in endothelial dysfunction in diabetes by generating reactive oxygen species [40]. Their increase could interfere with the phosphorylation of IRS-1/PI3K/Akt in the insulin signaling pathway, reducing insulin sensitivity, or promote the release of inflammatory factors from adipose tissue (such as TNF-α, IL-6), which further impair insulin signal transduction. In the current study, PC intervention decreased the content of the above BCAA metabolites, indicating that PC may alleviate T2DM by affecting the metabolism of glycated derivatives and their associated pathways. Moreover, according to our metabolic pathway prediction analysis, the increased abundance of the type 2 diabetes mellitus pathway in the model group indicates that our model was successfully established, and PC and MF interventions could decrease its abundance. PC intervention downregulated pathways associated with gluconeogenesis (e.g., glucagon signaling) and inflammation (e.g., HIF-1 signaling), while upregulating insulin secretion and p53 signaling, a key pathway for glucose homeostasis and cell survival [41]. PC intervention also increased the abundance of enzymes, especially K04962 (RYR2), involved in calcium signaling and insulin secretion, and K11533 (FAS), linked to fatty acid synthesis, which may improve lipid metabolism. Conversely, PC reduced enzymes annotated in the pentose and glucuronate interconversions pathway (e.g., K01812, K00854), which is dysregulated in T2DM [42]. Correlation analysis revealed that PC-enriched genera *SC-I-84* and *Bifidobacterium* positively correlated with beneficial metabolites (e.g., TUDCA) and enzymes (K11533), while harmful genera (*Mucispirillum*, *Desulfovibrio*) correlated with pro-inflammatory metabolites and dysregulated enzymes. These associations suggest that PC-induced changes in gut microbiota may be linked to altered metabolite profiles and predicted enzyme functions, but direct causal relationships need further analysis. The pathway enrichment and KEGG topological analyses indicated that PC’s anti-T2DM mechanism is closely associated with the regulation of bile acid, BCAA, and lipid metabolites, which could control host FBG and insulin levels through modulation of the insulin secretion and related pathways. Although these correlations provide potential mechanistic clues, they do not prove that changes in *SC-I-84*, *Bifidobacterium*, TUDCA, or LysoPC(P-16:0/0:0) directly mediate the metabolic effects of PC. Future studies using germ-free models, fecal microbiota transplantation, or targeted metabolite interventions would be needed to establish causal relationships.

## 5. Conclusions

In summary, intervention with the PSPA-derived anthocyanin fraction PC was associated with reduced water intake in T2DM rats, whereas its effects on fasting serum glucose and certain lipid biomarkers were not statistically significant under the current experimental conditions. PC intervention was also associated with lower insulin levels, although differences between groups did not reach statistical significance. PC modulated the gut microbiota, including enrichment of *Bifidobacterium*, and was associated with changes in related metabolites such as conjugated bile acids (e.g., TUDCA), BCAAs, and LysoPCs. Notably, TUDCA levels were higher in the PC group than in the MF group. Given that RyR2-mediated Ca^2+^ signaling has been implicated in insulin secretion and glucose homeostasis in pancreatic β cells, these microbiota-metabolite changes may be relevant to β-cell function; however, pancreatic histology and pancreatic enzyme activity were not assessed in this study, and the present data do not establish a direct effect on β-cell function. The observed microbiota-metabolite associations do not establish causality, and the potential link between PC-induced microbial changes and RyR2 expression remains to be elucidated. Therefore, larger studies are required for confirmation, incorporating transcriptomic, histological, and functional assessments.

## Figures and Tables

**Figure 1:**
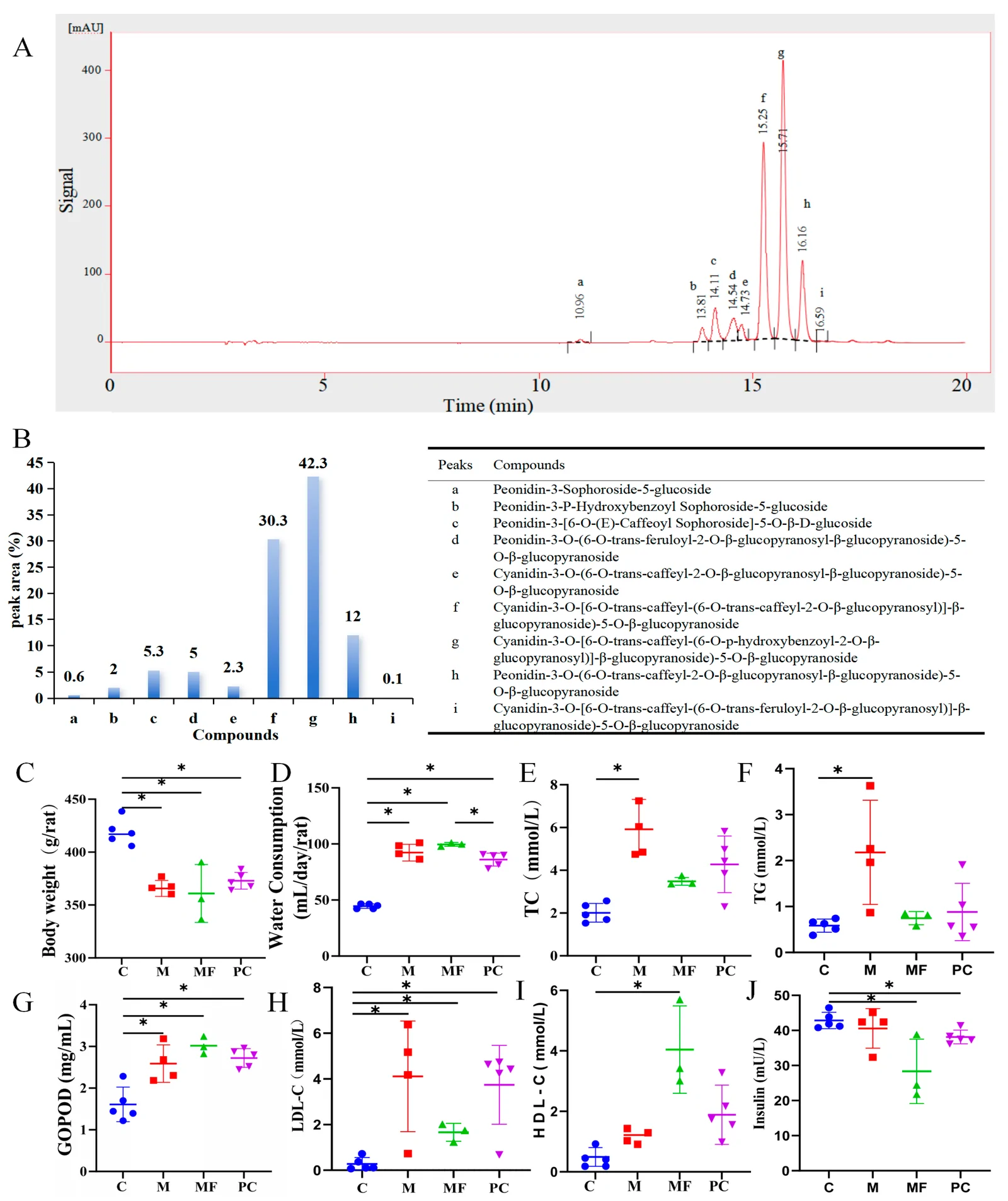
PC structure and its effect on biochemical indices: (**A**) HPLC monomer of PC component, (**B**) the component name and peak area information of PC, (**C**) body weight, (**D**) water intake, (**E**) serum TC, (**F**) serum TG, (**G**) serum GOPOD, (**H**) serum LDL-C, (**I**) serum HDL-C, and (**J**) serum insulin levels at the end of experiment. C: control group; M: T2DM model group (blue line/bar); MF: metformin intervention group; PC: paeonidin-and cyanidin-rich fractions (*n* = 5, 4, 3, and 5 rats for C, M, MF and PC, respectively). *, *p* < 0.05.

**Figure 2:**
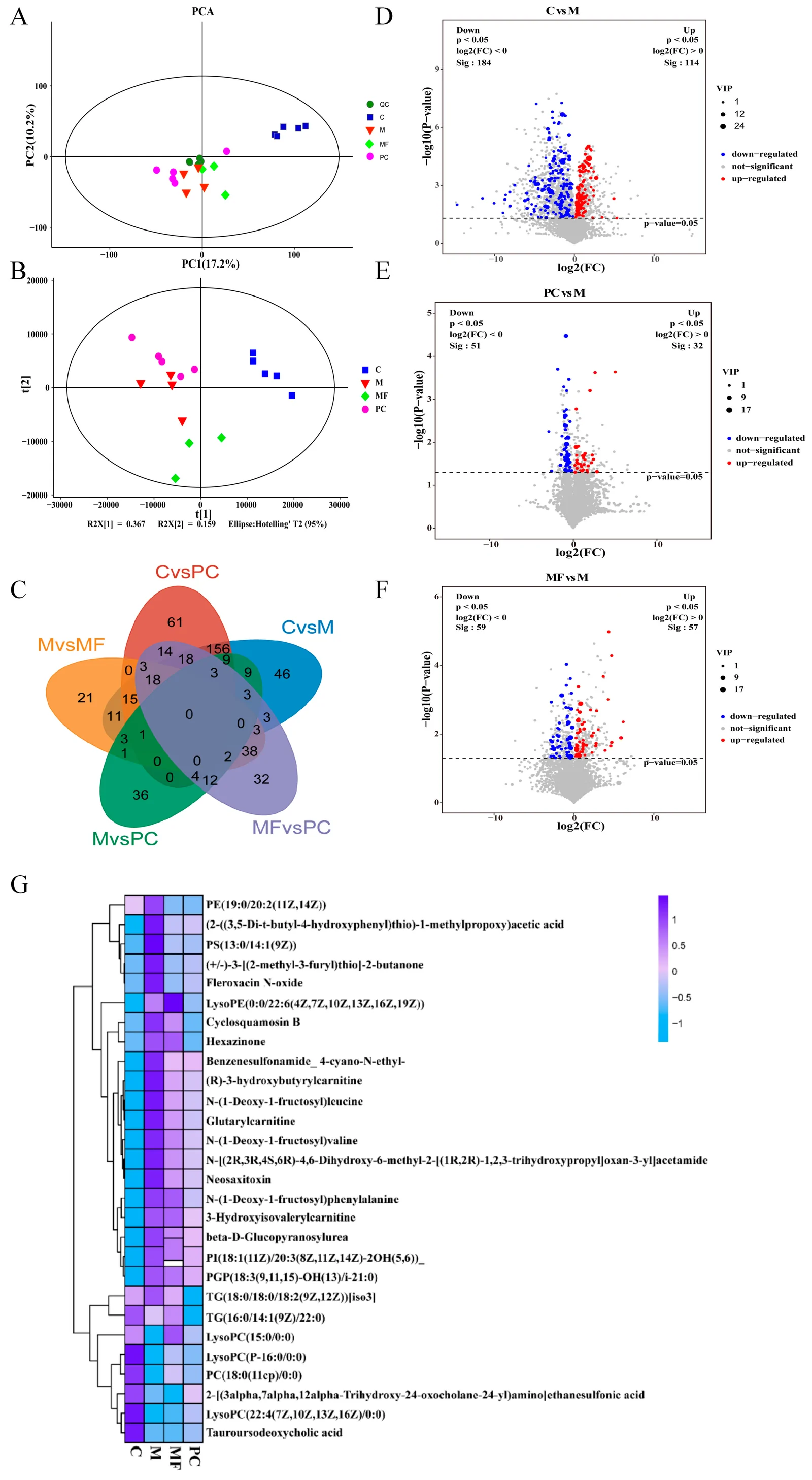
PC effects on serum metabolites. (**A**) PCA analysis; (**B**) metabolic changes by OPLS-DA analysis; (**C**) Venn diagram of significantly different metabolites across groups; (**D**) different metabolites between C and M; (**E**) different metabolites between MF and M; (**F**) different metabolites between PC and M group; (**G**) different metabolites within groups (*n* = 5, 4, 3, and 5 rats for C, M, MF and PC, respectively).

**Figure 3:**
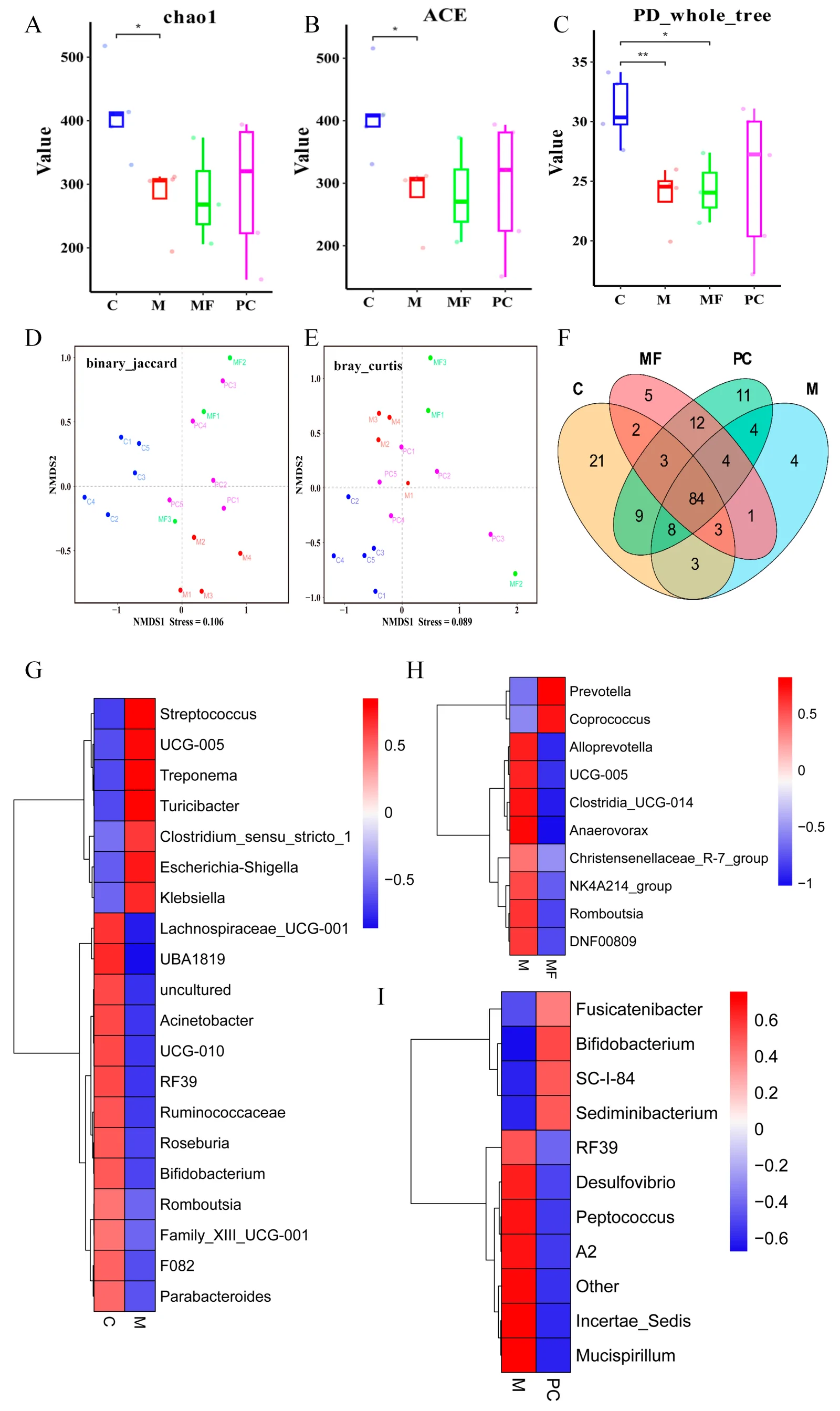
PC effect on gut microbial composition. (**A**) Chao1 α-diversity analysis; (**B**) ACE α-diversity analysis; (**C**) phylogenetic diversity of gut microbial; (**D**) binary jaccard beta diversity; (**E**) bray curtis beta diversity; (**F**) Venn diagram of significantly different genera among groups (ANOVA); (**G**–**I**) different bacteria genus between groups (*n* = 5, 4, 3, and 5 rats for C, M, MF and PC, respectively). *, *p* < 0.05; **, *p* < 0.01.

**Figure 4:**
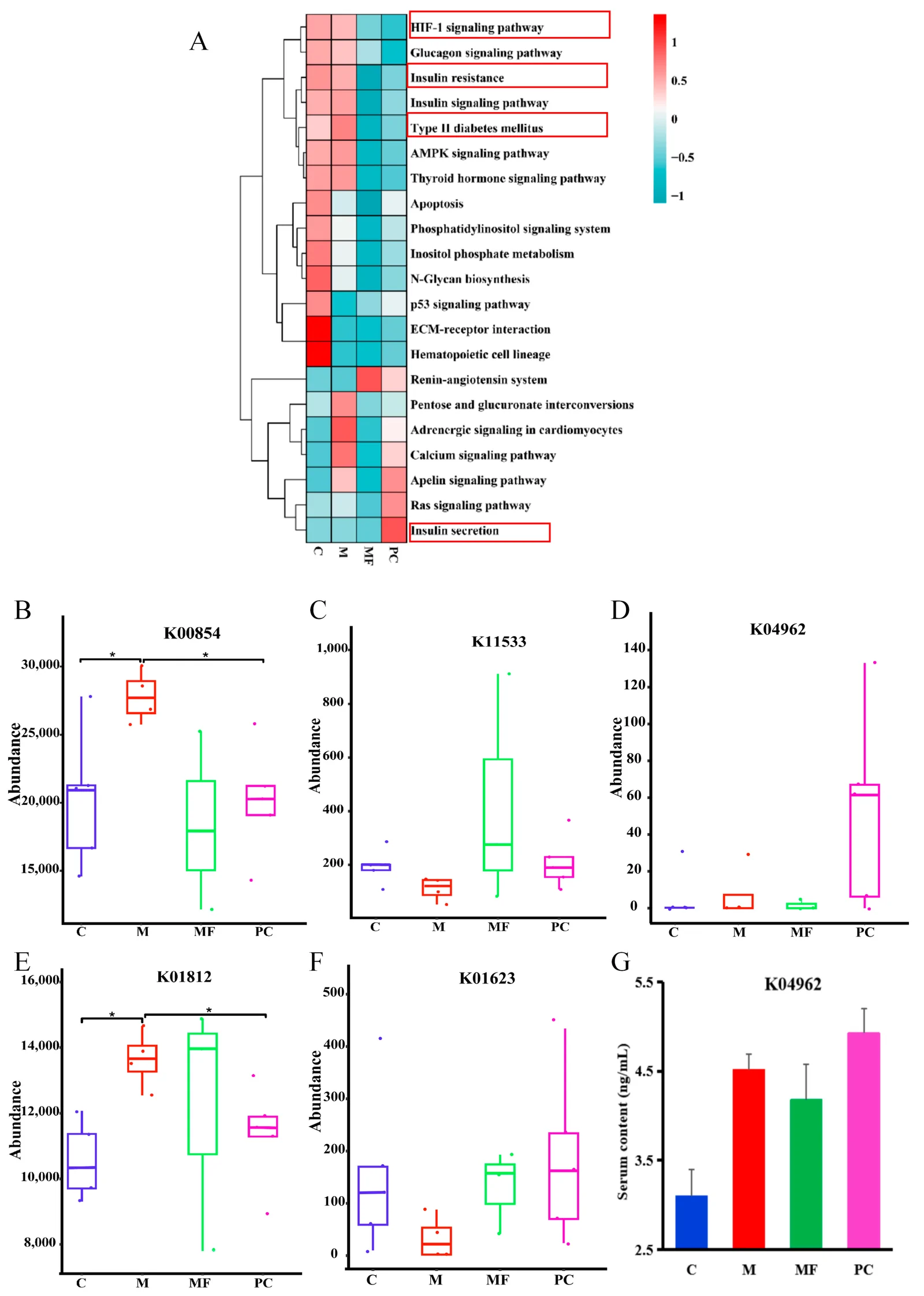
PC effects on gut microbial metabolic pathway. (**A**) Significantly different metabolic pathways within groups, red boxes indicate the previously reported pathways related to T2DM; (**B**–**F**) significantly different genes/enzymes; (**G**) serum K04962 content quantified by ELISA kit (*n* = 5, 4, 3, and 5 rats for C, M, MF and PC, respectively). *, *p* < 0.05.

**Figure 5:**
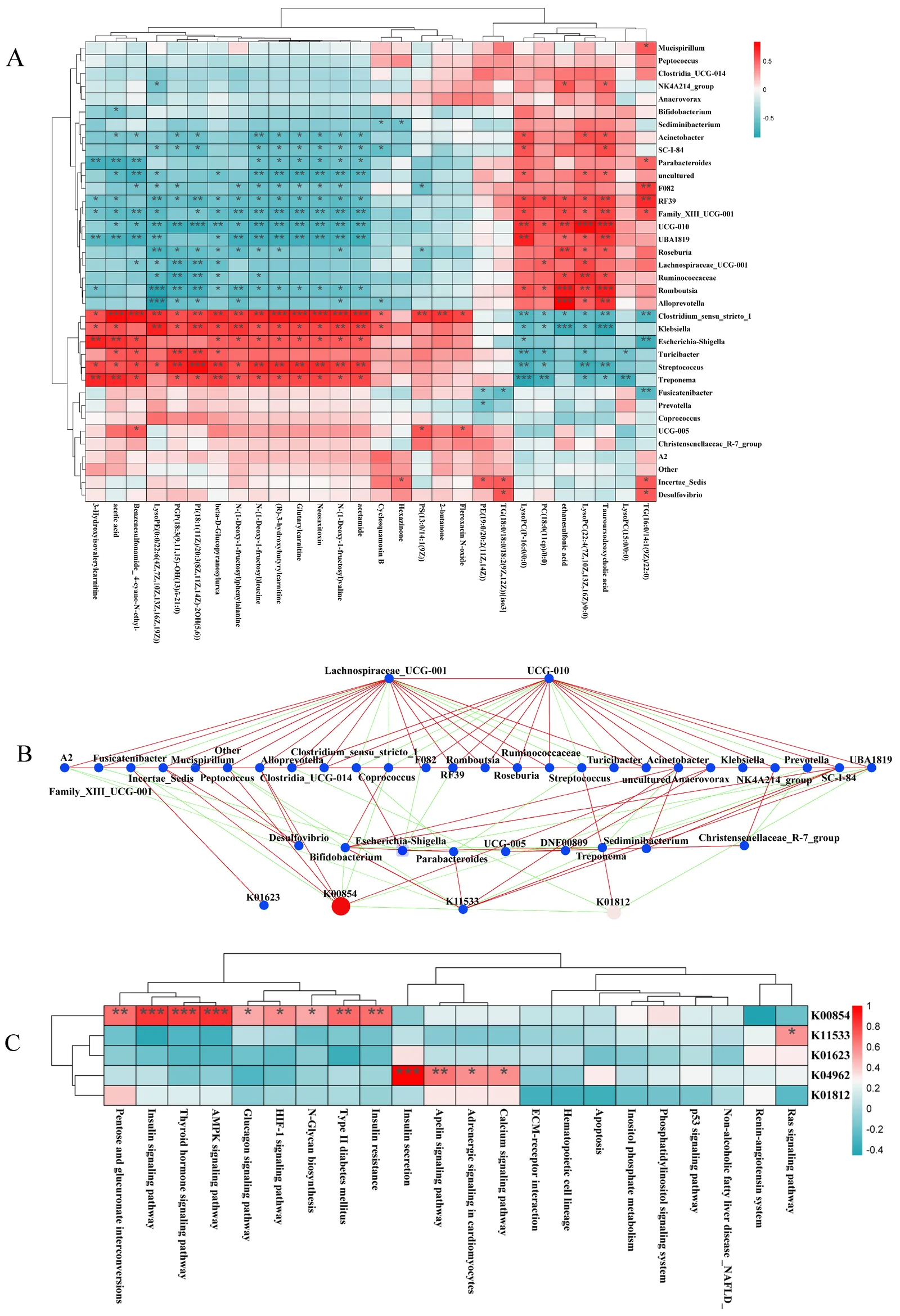
Correlation analysis. (**A**) correlation analysis between different genera and different metabolites; (**B**) correlation between different bacteria and different genes, dot color denotes relative abundance: red for high abundance, and blue for low abundance; line color denotes correlation type: red represent positive correlation, green represent negative correlation; (**C**) correlation between different metabolic pathways and different genes (*n* = 5, 4, 3, and 5 rats for C, M, MF and PC, respectively). *, *p* < 0.05, **, *p* < 0.01, ***, *p* < 0.001.

## Data Availability

The 16S rRNA sequencing data have been deposited in the SRA of the NCBI database. The Metabolomics data have been deposited in the EMBL-EBI MetaboLights database. Other data supporting this study’s findings are available in this article’s Supplementary Material.

## Supplementary Materials

The following supporting information can be downloaded at: https://www.mdpi.com/article/10.3390/nu18213520/s1, Table S1: Specific genus in each group; Table S2: Percentage of Super class Level significantly differential metabolites between groups.
