# Supplementary material for: Paeonidin- and Cyanidin-Rich Fractions Ameliorate Gut Microbiota and Serum Metabolites in Rats with Type 2 Diabetes
Source: Nutrients. 2026 Oct 26;18(21):3520. doi: 10.3390/nu18213520 (PMC13765502; doi:10.3390/nu18213520)
Supplement: Supplementary file 1 [file nutrients-18-03520-s001.zip › nutrients-4612386-supplementary.pdf]

Supplementary Table S1 Specific genus in each group.

| Groups | Specific Features | Relative Abundance |
|---|---|---|
| | *Tyzzerella* | 0.001591053638394 |
| | *Lachnospiraceae_NC2004_group* | 0.0012599884508583 |
| | *Barnesiella* | 0.0007575940849606 |
| | *Family_XIII_UCG-001* | 0.000656905756348133 |
| | *Staphylococcus* | 0.000410763005633181 |
| | *_Clostridium__methylpentosum_group* | 0.0003722690849289 |
| | *Anaeroplasma* | 0.0003687768899815 |
| | *Lachnospiraceae_FCS020_group* | 0.0002985336728422 |
| | *Actinomyces* | 0.0002225867813803 |
| | *IS-44* | 0.0001554028386918 |
| C | *MND1* | 0.0001189848889191 |
| | *Paracoccus* | 0.0001189848889191 |
| | *Nordella* | 0.0001036018924612 |
| | *Pseudonocardia* | 0.0001019870476449 |
| | *Actinomadura* | 0.0001019870476449 |
| | *Fenollaria* | 0.000101601923663 |
| | *_Anaerorhabdus__furcosa_group* | 0.000101601923663 |
| | *Globicatella* | 8.466826971924e-05 |
| | *Peredibacter* | 6.90679283074904e-05 |
| | *Leisingera* | 5.18009462306178e-05 |
| | *Christensenellaceae* | 3.62476439031463e-05 |
| | *Marinifilum* | 0.0001156451346439 |
| M | *Altererythrobacter* | 3.35750738651625e-05 |
| | *Candidatus_Soleaferrea* | 3.35750738651625e-05 |
| | *Candidatus_Stoquefichus* | 3.35750738651625e-05 |
| | *Dubosiella* | 0.0003414134516899 |
| | *Anaerostipes* | 0.0001437530322905 |
| MF | *Luteimonas* | 7.02049985959e-05 |
| | *Anseongella* | 3.510249929795e-05 |
| | *GOUTA6* | 3.510249929795e-05 |
| | *Cronobacter* | 0.0024097554664251 |
| | *_Eubacterium__hallii_group* | 0.0016305208951416 |
| | *Odoribacter* | 0.0010909408413252 |
| | *UCG-002* | 0.0008237685090921 |
| | *Erysipelotrichaceae_UCG-003* | 0.0006758434847615 |
| PC | *Oribacterium* | 0.0005013585198602 |
| | *Anaerosporobacter* | 0.0002189399936002 |
| | *Pseudomonas* | 0.0001132099883555 |
| | *MB-A2-108* | 6.46914219174537e-05 |
| | *Anaerofustis* | 6.46914219174537e-05 |
| | *Abditibacterium* | 4.85185664380903e-05 |

Supplementary Table S2: Percentage of Super class Level significantly differential metabolites between groups.

| Metabolites Super Class | C vs M | | MF vs M | | PC vs M | | MF vs PC | | PC vs C | |
|---|---|---|---|---|---|---|---|---|---|---|
| | No | (%) | No | (%) | No | (%) | No | (%) | No | (%) |
| Alkaloids and derivatives | 3 | 1.01% | 0 | 0% | 0 | 0% | 1 | 0.65% | 2 | 0.66% |
| Benzenoids | 12 | 4.03% | 7 | 6.03% | 4 | 4.82% | 7 | 4.58% | 12 | 3.97% |
| Lipids and lipid-like molecules | 144 | 48.32% | 39 | 33.62% | 40 | 48.19% | 68 | 44.44% | 159 | 52.65% |
| Nucleosides, nucleotides, and analogues | 5 | 1.68% | 1 | 0.86% | 1 | 1.20% | 3 | 1.96% | 7 | 2.32% |
| Organic acids and derivatives | 47 | 15.77% | 13 | 11.21% | 10 | 12.05% | 15 | 9.80% | 41 | 13.58% |
| Organic nitrogen compounds | 2 | 0.67% | 1 | 0.86% | 1 | 1.20% | 2 | 1.31% | 3 | 0.99% |
| Organic oxygen compounds | 21 | 7.05% | 10 | 8.62% | 9 | 10.84% | 15 | 9.80% | 23 | 7.62% |
| Organoheterocyclic compounds | 26 | 8.72% | 17 | 14.66% | 8 | 9.64% | 15 | 9.80% | 24 | 7.95% |
| Organosulfur compounds | 2 | 0.67% | 0 | 0% | 0 | 0% | 0 | 0% | 1 | 0.33% |
| Organohalogen compounds | 0 | 0% | 1 | 0.86% | 1 | 1.20% | 1 | 0.65% | 0 | 0% |
| Organophosphorus compounds | 0 | 0% | 1 | 0.86% | 1 | 1.20% | 0 | 0% | 0 | 0% |
| Phenylpropanoids and polyketides | 8 | 2.68% | 12 | 10.34% | 3 | 3.61% | 11 | 7.19% | 4 | 1.32% |
| Unclassified | 28 | 9.40% | 14 | 12.07% | 5 | 6.02% | 15 | 9.80% | 26 | 8.61% |
| Total | 298 | 100.00% | 116 | 100.00% | 83 | 100.00% | 153 | 100% | 302 | 100% |
